# Safety and implementation of phase I randomized GLA-SE–adjuvanted CH505TF gp120 HIV vaccine trial in newborns

**DOI:** 10.1172/JCI186927

**Published:** 2025-04-03

**Authors:** Avy Violari, Kennedy Otwombe, William Hahn, Shiyu Chen, Deirdre Josipovic, Vuyelwa Baba, Asimenia Angelidou, Kinga K. Smolen, Ofer Levy, Nonhlanhla N. Mkhize, Amanda S. Woodward Davis, Troy M. Martin, Barton F. Haynes, Wilton B. Williams, Zachary K. Sagawa, James G. Kublin, Laura Polakowski, Margaret Brewinski Isaacs, Catherine Yen, Georgia Tomaras, Lawrence Corey, Holly Janes, Glenda E. Gray

**Affiliations:** 1 Perinatal HIV Research Unit, Faculty of Health Sciences, University of the Witwatersrand, Johannesburg, South Africa.; 2 Vaccine and Infectious Disease Division, Fred Hutchinson Cancer Center, Seattle, Washington, USA.; 3 Department of Allergy and Infectious Disease, Division of Medicine, University of Washington, Seattle, Washington, USA.; 4 Department of Obstetrics and Gynaecology, Faculty of Health Sciences, University of the Witwatersrand, Johannesburg, South Africa.; 5 Precision Vaccines Program, Boston Children’s Hospital, Boston, Massachusetts, USA.; 6 Harvard Medical School, Department of Neonatology, Beth Israel Medical Center, Boston, Massachusetts, USA.; 7 National Institute for Communicable Diseases of the National Health Laboratory Service, Johannesburg, South Africa.; 8 South African Medical Research Council Antibody Immunity Research Unit, Faculty of Health Sciences, University of the Witwatersrand, Johannesburg, South Africa.; 9 Department of Integrative Immunobiology, Duke Human Vaccine Institute; and; 10 Department of Surgery, Human Vaccine Institute, Duke University School of Medicine, Durham, North Carolina, USA.; 11 Access to Advanced Health Institute, Seattle, Washington, USA.; 12 National Institute of Allergy and Infectious Diseases, Rockville, Maryland, USA.; 13 Center for Human Systems Immunology, Departments of Surgery, Immunology, and Molecular Genetics and Microbiology, Duke University, Durham, North Carolina, USA.; 14 South African Medical Research Council, Cape Town, South Africa.

**Keywords:** AIDS/HIV, Clinical Research, Vaccines, AIDS vaccine

## Abstract

**BACKGROUND:**

The neonatal immune system is uniquely poised to generate broadly neutralizing antibodies (bnAbs), and thus infants are ideal for evaluating HIV vaccine candidates. We present the design and safety of a new-in-infants glucopyranosyl lipid A–stable emulsion (GLA-SE) adjuvant admixed with a first-in-infant CH505 transmitter-founder (CH505TF) gp120 immunogen designed to induce precursors for bnAbs against HIV.

**METHODS:**

HIV Vaccine Trials Network 135 is a phase I randomized, placebo-controlled trial of CH505TF plus GLA-SE or placebo. Healthy infants aged ≤5 days, born to mothers living with HIV but HIV nucleic acid–negative at birth, were randomized to 5 doses of CH505TF plus GLA-SE or placebo at birth and 8, 16, 32, and 54 weeks.

**RESULTS:**

Thirty-eight infants (median age 4 days; interquartile range 4–4.75 days) were enrolled November 2020 to January 2022. Among 28 infants assigned to receive CH505TF plus GLA-SE and 10 assigned to receive placebo, most completed the 5-dose immunization series (32/38) and follow-up (35/38). Solicited local and systemic reactions were more frequent in vaccine (8, 28.6% local; 16, 57.1% systemic) versus placebo recipients (1, 10% local, *P* = 0.25; 4, 40.0% systemic, *P* = 0.38). All events were grade 1 except 2 grade 2 events (pain, lethargy). Serious vaccine-related adverse events were not recorded.

**CONCLUSION:**

This study illustrates the feasibility of conducting trials of new-in-infants adjuvanted HIV vaccines in HIV-exposed infants receiving standard infant vaccinations. The safety profile of the CH505TF plus GLA-SE vaccine was reassuring.

**TRIAL REGISTRATION:**

ClinicalTrials.gov NCT04607408.

**FUNDING:**

National Institute of Allergy and Infectious Diseases of the NIH under grants UM1AI068614, UM1AI068635, and UM1AI068618.

## Abstract 2

This paper summarizes the phase 1 trial design and safety profile of an experimental CH505TF immunogen + GLA-SE HIV vaccine in infants born to mothers living with HIV.

## Introduction

Standard clinical development for vaccines with potential applications in infants generally involves early-phase evaluations in adults, followed by evaluation in older children, and eventually neonatal populations — also called the age de-escalation approach; however, this process can take decades. In the case of preventive HIV vaccines, early evaluation in newborns and infants is supported by the following rationale: (a) growing literature suggests that owing to a distinct immunity profile in early life, natural HIV acquisition or immunization may more readily and rapidly induce broadly neutralizing antibodies (bnAbs) in infants than in adults (1–3); (b) infants may be an ideal population for current vaccine strategies that will likely require multiple doses administered at long intervals before protective immune responses are achieved, because, despite an initial period of risk of HIV acquisition associated with breastfeeding, infants are likely to have a long period with low risk of HIV exposure before sexual debut; and (c) infant populations already receive many vaccinations throughout the first year of life as part of standard of care, reducing the downstream operational complexities of implementing a potentially complex bnAb vaccine regimen.

The Antibody Mediated Prevention (AMP) trials (HIV Vaccine Trials Network [HVTN] 703/HIV Prevention Trials Network [HPTN] 081 and HVTN 704/HPTN 085) provide proof that bnAbs can prevent HIV infection (4). Given this, we reason that neonatal populations should be integrated into the early clinical development plan of preventive HIV vaccines because vaccine candidates that might not elicit bnAbs in adults or older children could plausibly do so in infants (1–3). Furthermore, vaccine immunogenicity and safety in early life are not directly translatable from adult studies. While the neonatal immune system has been traditionally described as “immature,” or naive, current understanding is that it is “distinct,” characterized by an initial fetal and maternal immune tolerant state with CD4 Th2-polarized responses and underdeveloped germinal centers (5), such that immune responses to pathogens and vaccines differ from those of adults. A broad range of relative immunogenicity is observed in newborns compared with older children and adults for various vaccines. For example, pneumococcal polysaccharide vaccine is not immunogenic in children less than 2 years of age (6), the DTaP vaccine exhibits reduced immunogenicity (7), and the pneumococcal conjugate vaccine (PCV) has demonstrated comparable binding antibody responses with higher avidity (8, 9). Indeed, some measures of antibody quality, such as avidity, were higher in infants vaccinated with PCV7 at birth when compared with standard Expanded Program on Immunization (EPI) vaccine schedules beginning at 6 weeks of age (8).

HVTN 135 was designed to assess the HIV envelope CH505 transmitter-founder (CH505TF) immunogen adjuvanted with glucopyranosyl lipid A–stable emulsion (GLA-SE) in neonates. The immunogen was designed to induce antibody lineages for the CD4 binding site that have the potential to develop neutralization capacity. The addition of a new-in-infants adjuvant may be central to enhancing vaccine immunogenicity in vulnerable populations, including newborns and young infants. Many early studies use Alum, the most common vaccine adjuvant included in most licensed pediatric vaccines. Although Alum is generally safe, there are known limitations to the effectiveness of this Th2-polarizing adjuvant in infant populations (10). GLA-SE is a synthetic TLR4 agonist formulated in a nano-emulsion of squalene oil, is Th1 polarizing, and was shown to increase antibody avidity and breadth when compared with Alum in infant macaques (10). A key goal of this study was to establish the safety of this adjuvant in human infants.

Conducting early-phase clinical trials in neonatal populations is logistically challenging because of the inherent vulnerability of this population. Infants exposed to HIV are at higher risk for poor outcomes relative to neonates who are not exposed (11), most often because of other concurrent infections, particularly lower respiratory tract infections, gastroenteritis, and sepsis. Establishing a framework to conduct early-phase vaccine trials in a safe and ethical manner in this vulnerable population is therefore of critical importance. To this end, a team of investigators, community representatives, and other stakeholders convened to design and conduct a phase I clinical trial to test the safety, tolerability, and immunogenicity of a new-in-infants GLA-SE–adjuvanted CH505TF gp120 vaccine in healthy newborn infants exposed to HIV and living without HIV. Additional exploratory endpoints include in vitro modeling of adjuvant and antigen action as well as systems vaccinology to define biomarker correlates of vaccine action, approaches consonant with the recent FDA Modernization Act 2.0 (12). Here we report the safety data and provide insights regarding preparatory activities, study design elements, and implementation considerations from this phase I trial, which is to our knowledge the first of its kind conducted in the last decade among infants exposed to HIV who did not acquire HIV.

## Results

**Figure 1:**
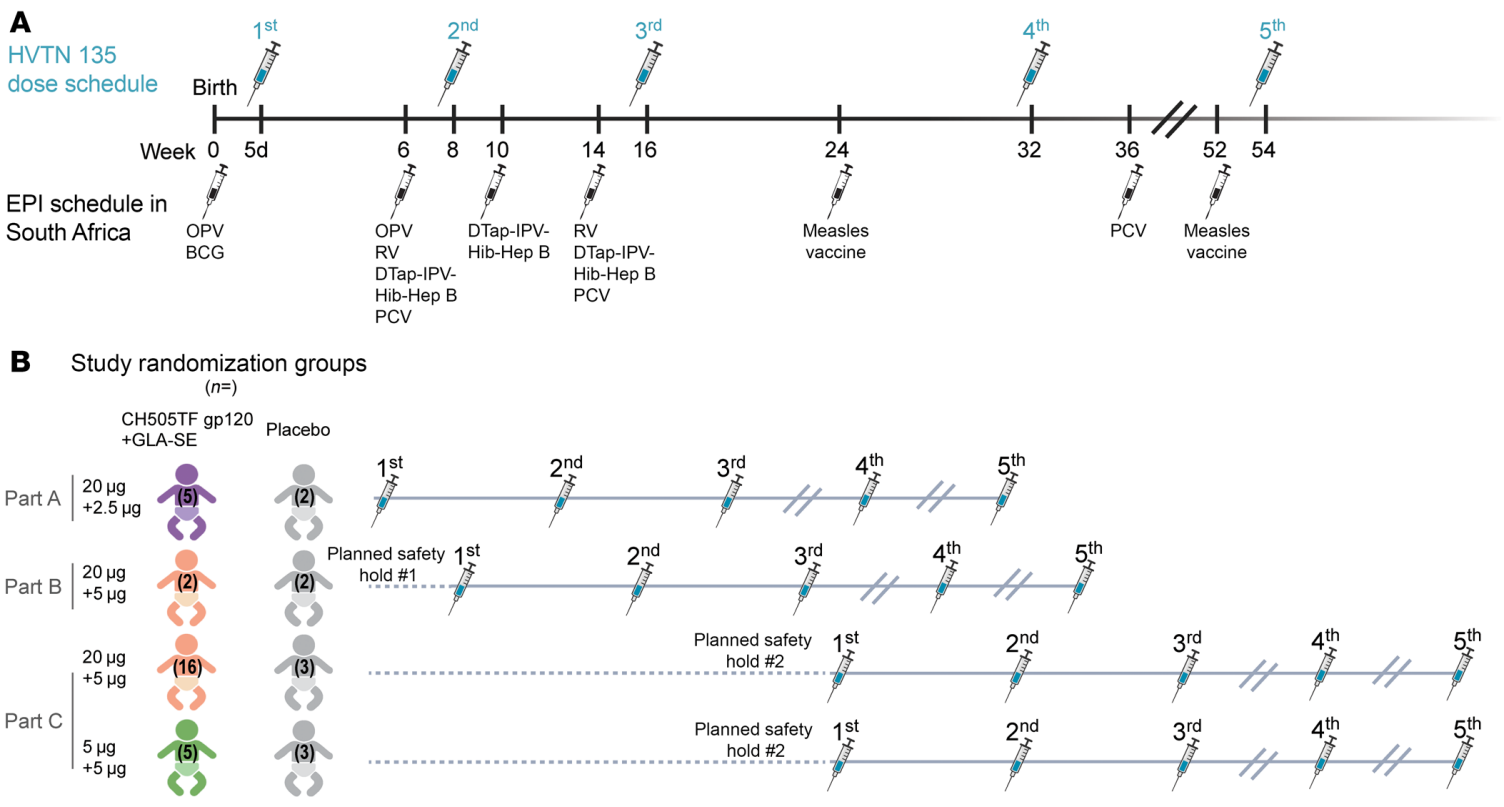
Study design. (**A**) Vaccine dosing schedule in relation to EPI vaccinations. (**B**) Study schema: Healthy infants without HIV born to mothers living with HIV were randomized to receive 5 doses of CH505TF gp120 plus GLA-SE vaccine or placebo, with the first dose within 5 days of birth. The 3-part design allowed for an initial assessment of safety in part A before the target adjuvant dose was studied in part B. Part C compared 2 different doses of CH505TF gp120 while keeping the same adjuvant dose.

A 3-part randomized, placebo-controlled study of the CH505TF gp120 immunogen with GLA-SE adjuvant was conducted in healthy newborn infants born to mothers living with HIV, enrolled within 5 days of birth. In each part, participants were randomized to receive 5 doses of the vaccine or placebo, at weeks 0, 8, 16, 32, and 54. The vaccination schedule was designed to minimize the overlap with routine infant immunizations (Figure 1).

### Demographics and baseline characteristics.

**Figure 2:**
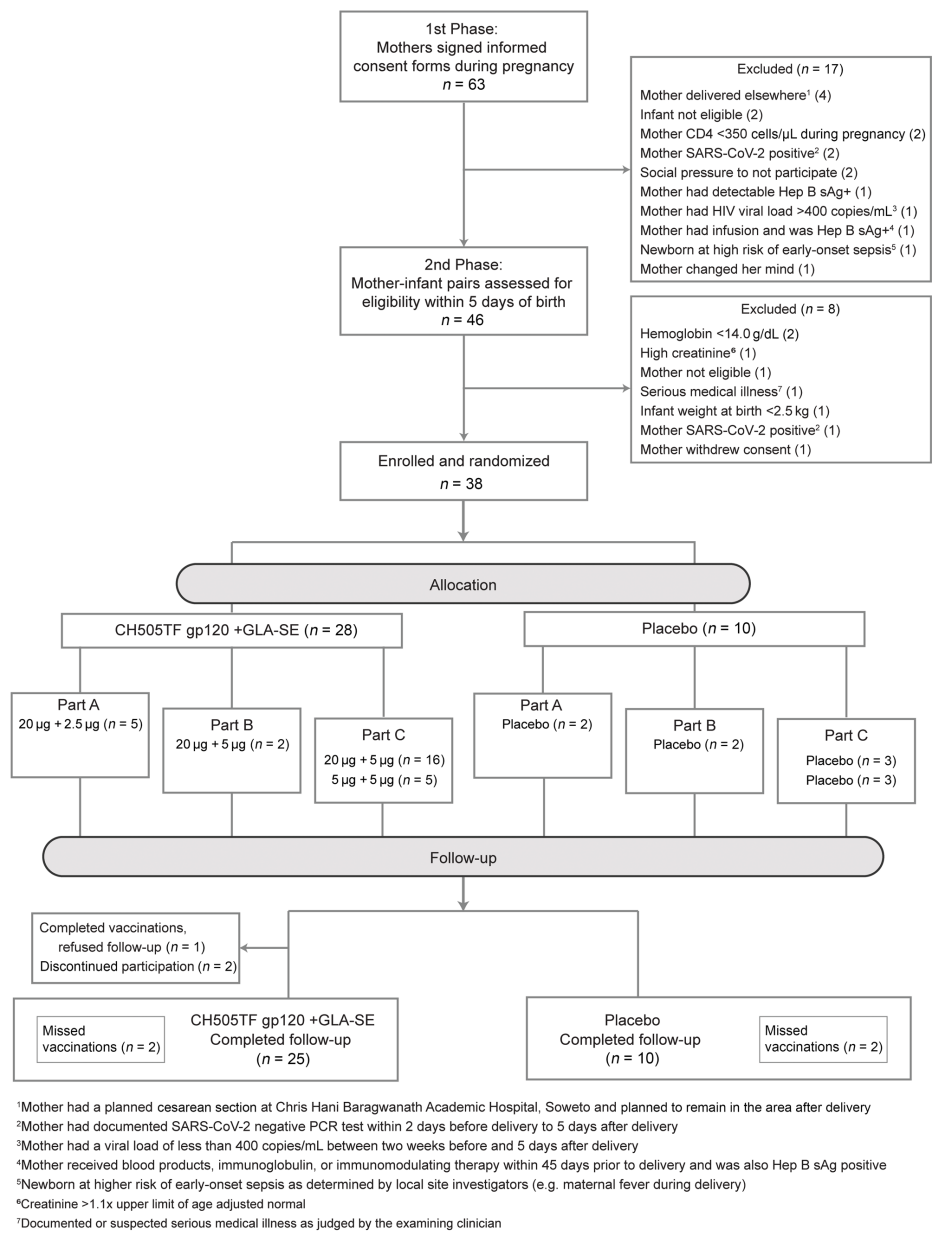
CONSORT diagram. Flow of participants, from screening to randomization, enrollment, follow-up, and study completion.

Among 63 mothers who provided consent for themselves and gave permission for their infants to participate in the study, 46 were assessed for eligibility within 5 days of birth (17 became ineligible during pregnancy), and 38 mother/infant pairs were enrolled (Figure 2). Most screening failures were due to maternal ineligibility (17 of 25 who were not enrolled), the most common reasons being acute medical incidents, including COVID-19 (Figure 2).

Of 38 infants enrolled, 18 (47.4%) were female (Table 1). Median age at first dose of study product was 4 days. The median (interquartile range) infant weight at enrollment was 3,035 (2,742.5, 3,377.5) g, and all were receiving antiretroviral therapy prophylaxis. Most infants were breastfed at birth (63.2%), and this declined to 33% by 13 months (Supplemental Table 1; supplemental material available online with this article; https://doi.org/10.1172/JCI186927DS1). The majority of mothers (71%) were between 31 and 40 years of age; 45% had some secondary/high school education, and 40% had completed secondary/high school. All mothers had a viral load below 400 copies/mL, while 95% had a viral load below the lower limit of detection for the assay used (Supplemental Table 2). Maternal and infant baseline characteristics were well balanced between vaccine and placebo groups.

### Compliance and retention.

Study retention was robust (Supplemental Table 3). Of the 38 infants enrolled, 35 completed follow-up. For 1 vaccine recipient, after completing vaccinations the caregiver refused follow-up. Another vaccine recipient was lost to follow-up when the caregiver relocated after the second vaccination, and the caregiver of a third vaccine recipient refused further participation for the infant after the third vaccination. Receipt of the 5-dose regimen was complete for 32 of 38 participants (24 of 28 vaccine recipients; 8 of 10 placebo recipients; Figure 2). Of the 6 participants who did not receive all 5 doses, 2 vaccine recipients mentioned above missed doses because of early study termination, while the other instances were due to missed visits during the study. None of the vaccine recipients missed vaccinations or terminated from the study early because of study product–related adverse events (AEs). The viral load of mothers was assessed periodically for the first year of life, and no mother had detectable viremia.

### Safety.

**Figure 3:**
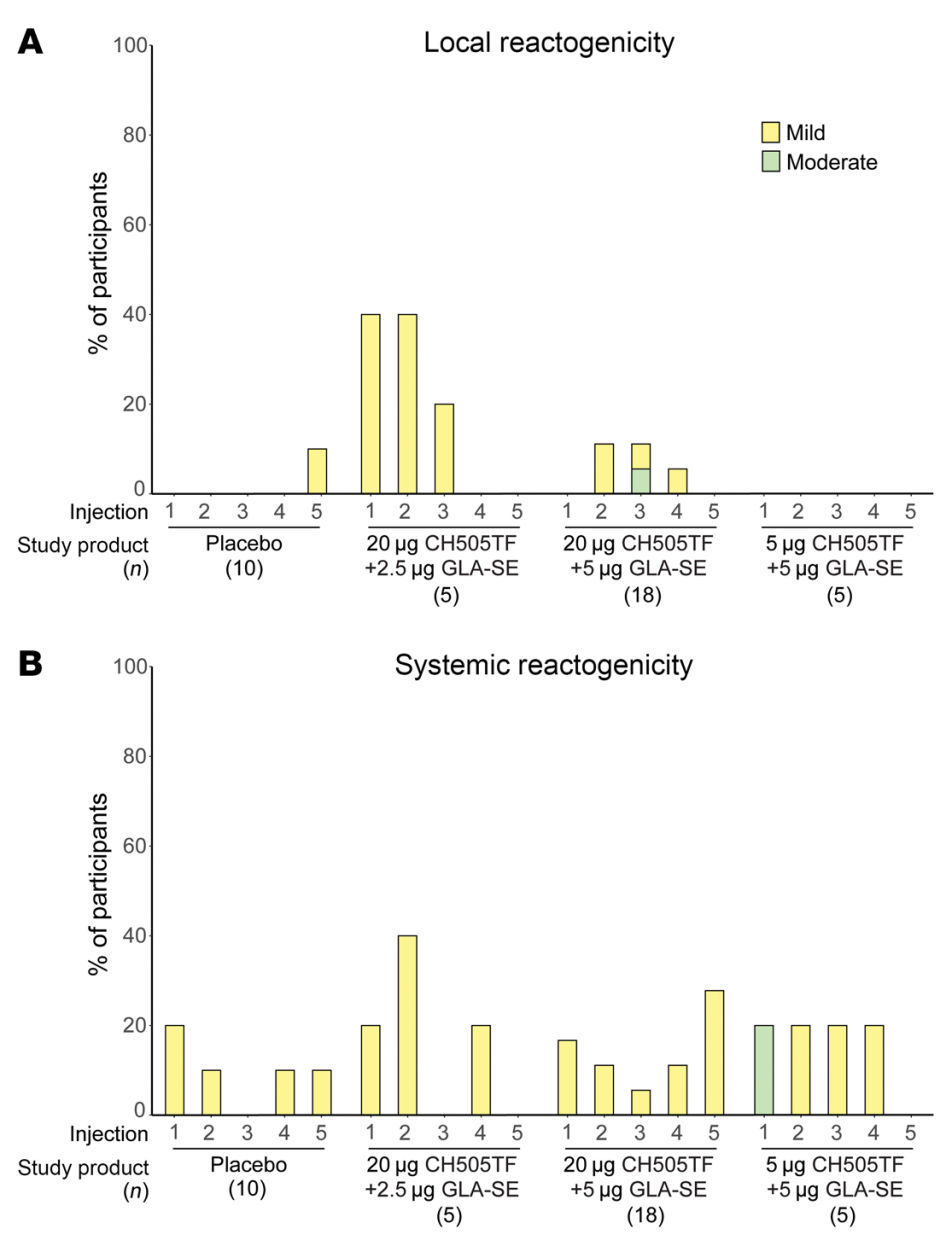
Local and systemic reactogenicity. Percentage of participants who experienced each grade of local and systemic reactogenicity by arm.

The vaccine demonstrated an acceptable safety profile and was well tolerated (Figure 3, Supplemental Table 4, and Supplemental Figure 1). Both local and systemic reactogenicity related to the vaccine was typically mild. Among all participants, the most commonly solicited AEs were sleepiness/lethargy (23.7%) and rash (26.3%), while mild fever was seen in 15.8%. One moderate-severity event of pain/tenderness occurred in the 20 μg CH505TF gp120, 5 μg GLA-SE group after the third dose, and 1 moderate sleepiness/lethargy event occurred in the 5 μg CH505TF gp120, 5 μg GLA-SE group after the first dose. Neither required medical intervention. No severe or life-threatening systemic or local solicited AEs were observed. Local reactogenicity rates were higher, but not statistically significantly different, in vaccine versus placebo groups (28.6% vs. 10%; *P* = 0.25), whereas the rates of systemic solicited AEs were more similar between groups (57.1% vs. 40%; *P* = 0.38).

Unsolicited AEs were generally mild or moderate in severity and consistent with common childhood illnesses (Supplemental Table 5). The most frequent AEs were gastrointestinal infections and hematologic abnormalities, both of which occurred more frequently in the placebo group (Table 2). Six AEs that met seriousness criteria were reported: 3 in the placebo group and 3 in the vaccine group (Table 3), all of which were deemed unrelated to the study product. Only 1 unsolicited AE (a grade 1 decreased neutrophil count) was deemed related to study product; this occurred in the placebo arm (Supplemental Table 6). Decreased neutrophil count *not* deemed related to study product was observed in all groups with approximately equivalent frequencies in each group and the highest numerical rate in the placebo arm (6/10 participants). None of the enrolled infants acquired HIV based on a Clinical Laboratory Improvement Amendments–approved PCR test through a minimum of 711 days of life.

### Early infant immunizations.

Bacille Calmette-Guérin (BCG), rotavirus (RV), DTaP-IPV-Hib-HBV, and PCV vaccination rates were high (>97%) through the first 9 months of life (Supplemental Table 3). OPV vaccine receipt was lower in frequency at birth (79%) and 6 weeks (84%). Ninety-seven percent of infants received measles vaccination at 12 months, and 91% received DTaP-IPV-Hib-HBV at 18 months. The most common reason for missed vaccinations was out-of-stock product (88% of the 69.5% of missed vaccinations for which a reason was provided).

### Infant growth.

At enrollment, 37 of 38 infants (97.4%) and 35 of 38 (92.1%) had weight-for-age and weight-for-length *z* scores, respectively, within 2 standard deviations of the population mean (*z* scores between –2 and 2). Healthy growth was observed in all infants over the 2 years of follow-up, with weight-for-age consistently within 2 standard deviations of the mean, and average weight-for-age trajectories following the population mean (Supplemental Figure 2). Vaccine and placebo weight-for-age and weight-for length averages were similar.

## Discussion

Conducting vaccine trials in a neonatal population presents substantial scientific, ethical, and operational challenges. HVTN 135 provides reassuring safety data for the studied HIV vaccine that includes a new-in-infants TLR4 agonist adjuvant (GLA-SE) and demonstrates the feasibility and safe implementation of a phase I study in neonates vaccinated within 5 days of birth. Additionally, the study demonstrates the feasibility of collecting cord blood samples that can be used for planned cellular analysis in the context of an HIV vaccine trial.

The safety and tolerability of the candidate vaccine were reassuring. There were only 2 moderate (grade 2) vaccine reactogenicity events, 1 for pain/tenderness at the injection site in the 20 μg CH505TF gp120, 5 μg GLA-SE group and another for sleepiness/lethargy in the 5 μg CH505TF gp120, 5 μg GLA-SE group. All other reactogenicity events were mild. Overall, 61% of participants experienced a reactogenicity event. All unsolicited AEs were deemed not related to study product, except for 1 grade 1 neutropenia in the placebo group, which had been assessed as related before unblinding. Neutropenia was common in our study with an overall rate of 34% (60% in the placebo group and 25% in the vaccine group) and was primarily attributed to cotrimoxazole prophylaxis, which was part of the standard of care for infants exposed to HIV at the time of enrollment. Mild neutropenia is a commonly observed laboratory abnormality in this population, with high rates of neutropenia occurring in previous vaccine trials in infants exposed to HIV mostly attributed to concomitant medications such as cotrimoxazole (13).

Neonatal and infant vaccination is one of the most important interventions in child health — often delivered at scale even in resource-poor settings within a primary health care clinic infrastructure. This study opens the possibility for similar vaccine trials against HIV and other pathogens in this population. Further studies of candidate HIV vaccines with similarly acceptable safety and feasibility would provide support for including infants in trials at a much earlier stage in vaccine development, thereby accelerating vaccine development and enhancing vaccine availability for younger populations.

Choosing infants exposed to HIV as the study population has several advantages and disadvantages. The same population has been involved in previous HIV vaccine studies, and it is conceivable that acceptability of such a study may be higher in families affected by HIV. However, interpretation of immune responses to the vaccine can be more challenging because of the presence of maternal antibodies (anti-gp120 and anti-CD4bs), which are present for at least 6 months. Coenrolling the mother in the study and including a placebo arm facilitate interpretation of the results.

Any phase I study in this vulnerable age group should consider input from experts in neonatal immunology and vaccinology as well as include consultation with ethicists and community advisory members. Our study was successful in incorporating community feedback throughout protocol development; importantly, this began very early during the protocol design phase. By engaging with the community early in the process, we built trust and maintained open channels of communication, which resulted in robust recruitment and retention. For example, in addition to the care related to vaccination, the community team highlighted that an open-door policy with access to routine pediatric care as needed would also be an important element for participation in the study for women and their infants. The community team also engaged community members regarding the acceptability of cord blood donation. We would recommend that other early-phase vaccine studies use similar methods to ensure community involvement.

Limiting inclusion to cesarean deliveries allows for collection of cord blood, allowing for in-depth immunologic testing and sparing the amount of blood taken from the baby at birth. It is not a biological requirement of the vaccine, and the population would be expanded if the vaccine is advanced in clinical development. Even in the context of an early-phase study, however, it could slow down enrollment either by limiting the pool of mothers available for recruitment or when participants deliver vaginally prior to the scheduled cesarean delivery, as was the case in our study. Planned cesarean delivery in low-resource areas can often take place outside the scheduled date owing to multiple obstetric emergencies that take precedence. This requires the study team and the laboratory receiving the specimens to be on standby and available at night, which requires resources and logistics that in the case of our study were further complicated by the COVID-19 pandemic and subsequent frequent restrictions at operating theaters.

An important consideration regarding our study is that healthy neonates exposed to HIV and living without HIV have a higher rate of viral infections and have a higher burden of serious health outcomes than their HIV-unexposed counterparts (14, 15). Including HIV-exposed infants in a clinical trial with a rigorous structure for ensuring participant safety entailed careful monitoring of infant growth rate as well as key health outcomes. Reassuringly, we found that the infants in our study developed at a rate within the normal range. By including details of the protocol conduct, we provide the wider pediatric community with insight into the operational details of a neonatal vaccine trial in a population of infants exposed to HIV. Another benefit of our trial is that robust, high-quality safety data from the placebo arm can help inform the design of future studies in this population.

There are several limitations of our study related to the study population. Because of the desire to pursue detailed immunologic assessments requiring large blood volumes, the initial population was restricted to infants born via cesarean delivery, which is more amenable to collection of cord blood compared with vaginal delivery. Additionally, infants were born to mothers living with HIV such that results may not be directly applicable to a broader population without HIV. Additional safety and immunogenicity data would likely be required before inclusion of these populations in a potential efficacy trial. Another potential limitation is that we separated the study product from standard EPI vaccines. This design was selected to enhance detection of any study vaccine–associated safety signals. If such an infant HIV vaccine advances to a large-scale efficacy trial, it would be more practical to administer it concomitantly with conventional EPI vaccines. Given the possibility of vaccine-vaccine interactions, additional bridging studies may be required to evaluate the effect of coadministration on the safety or immunogenicity of the study vaccine and/or EPI vaccines.

During protocol development it was also critical to factor in that there are substantial limitations on sampling that can be performed with infants, especially with respect to blood volumes. Substantial limitations on blood volumes require careful planning for the immunologic specimens that can be collected and careful monitoring for iatrogenic anemia. To make optimal use of the small sample volumes available, we leveraged a potentially novel sample sparing workflow (16). Ongoing work will examine molecular signatures underlying the observed immunogenicity effects using modern systems vaccinology approaches. Evaluation of the immunogenicity of the current vaccine is also ongoing, including a planned comparison of infants in HVTN 135 with adults in HVTN 115 receiving the same GLA-SE–adjuvanted gp120 protein vaccine, which will offer unique insight into the adult versus infant immune response to this vaccine. As the vaccine had a very reassuring safety profile, we are planning to report the full immunogenicity results, which we believe will support the proposition that GLA-SE is an attractive adjuvant in the neonatal population. Human in vitro modeling has also been leveraged to characterize the action of adjuvant and antigen toward leukocytes from the same participants studied in vivo (17). Despite the challenges of conducting research in a neonatal population, there is an urgent need to study the molecular mechanisms of vaccine safety and immunogenicity in this age group, which has distinct immunologic features and receives many vaccines.

In addition to the expected scientific insights, HVTN 135 serves as an example for integrating neonatal vaccines early into the vaccine development plan, and its operational conduct provides a template for future vaccine trials conducted in this population, including additional pediatric HIV vaccine trials. A successful neonatal vaccine could be given to all infants irrespective of whether their birth parent is living with HIV, supporting a continuum of protection from early life to sexual debut.

## Methods

### Sex as a biological variable

Our study examined male and female infants, and no differences in safety profile were detected between the sexes.

### HVTN 135 study design

#### Study population.

Participants were healthy newborn infants exposed to HIV who were born to mothers living with HIV, enrolled within 5 days of birth. Key inclusion criteria for the pregnant women were well-controlled HIV, as demonstrated by virologic suppression (HIV viral load of less than 400 copies/mL) and a CD4 count greater than 350 cells/μL, and planning to have cesarean delivery. Planned cesarean delivery was required because of the need to collect cord blood, which is logistically challenging in the setting of spontaneous vaginal delivery. Key inclusion criteria for the infant included a birth weight above 2.5 kg, estimated gestational age greater than 37 weeks, a negative HIV nucleic acid test at birth, and antiretroviral prophylaxis consistent with the local standard of care.

#### Study investigational products.

The CH505TF gp120 immunogen was derived from a clade C transmitted/founder HIV virus isolated from a single acutely HIV-infected donor (CH505) (18–20). The adjuvant GLA-SE is an oil-in-water stable emulsion containing the immunologic adjuvant glucopyranosyl lipid A (GLA). CH505TF gp120 is manufactured by Berkshire Sterile Manufacturing, and GLA-SE is manufactured by the Infectious Disease Research Institute, Seattle, Washington, USA; both products were provided by the Division of AIDS (DAIDS) of the National Institute of Allergy and Infectious Diseases of the US NIH.

#### Design and rationale.

A 3-part, randomized, placebo-controlled design was used (Figures 1 and 2). A placebo group was deemed important to aid unbiased reporting and interpretation of safety events. Given that infants who are exposed to HIV acquire HIV-specific immune responses from their mothers, the inclusion of a placebo arm also allowed differentiation of vaccine-induced versus maternally derived immune responses, by virtue of vaccine versus placebo comparisons. Specimens were collected throughout the first 2 years of life, as maternally derived antibodies are anticipated to wane about 6 months after birth. The 3-part design minimized exposure to higher doses of the adjuvant until safety data with lower doses were collected. Part A of the study provided initial safety data on the CH505TF immunogen (20 μg) when combined with a 2.5 μg dose of the GLA-SE adjuvant. Part B provided a preliminary safety assessment of the maximum targeted 5 μg dose of the GLA-SE adjuvant, since this dose was under concurrent evaluation in separate non–HIV vaccine pediatric studies (ClinicalTrials.gov NCT03806699 and NCT03799510). Enrollment into part B began after the study’s first planned safety hold and review of the cumulative safety data for all participants in part A up to and including 2 weeks following the first dose of study product (i.e., CH505TF+GLA-SE or placebo). Part C expanded the evaluation of the 20 μg CH505TF/5 μg GLA-SE regimen and also included for comparison a dose-sparing 5 μg CH505TF plus 5 μg GLA-SE arm. Enrollment into part C began after a second planned safety hold and review of cumulative safety data for all participants in part A and for all participants in part B up to and including 2 weeks following the third dose of study product.

In each part, participants were randomized to receive 5 doses of the vaccine or placebo, at weeks 0, 8, 16, 32, and 54. This schedule was designed to minimize overlap with the Republic of South Africa (RSA) EPI vaccination schedule (Figure 2A). If an infant was found to be delayed in EPI vaccines at a study visit, priority was given to catching up on the EPI vaccines before administering study product. EPI vaccines were given at least 7 days before administration of study vaccination, and EPI vaccine immune responses will be evaluated in all infants.

#### Study procedures.

Participants received study product at the indicated doses via 0.25 mL or 0.5 mL injections administered intramuscularly in the thigh. Both the placebo and the diluent were 0.9% sodium chloride. All participants were observed for a minimum of 60 minutes after study product administration.

For 7 days after each study product injection, caregivers recorded predefined solicited AEs (also known as “reactogenicity”) using a participant diary. Unsolicited AEs were collected throughout the main study and coded per the MedDRA and graded for severity according to the DAIDS Table for Grading of the Severity of Adult and Pediatric Adverse Events, version 2.1. Participants were followed for 12 months after the final study product dose, through approximately 2 years of life, with a total of 14 in-person visits.

Protocol-required samples included cord blood and peripheral infant blood specimens for safety and immunogenicity analyses. Optional samples included maternal breast milk and stool from both mother and infant. Infant anthropometric measurements were obtained at each study visit to monitor infant growth as part of ongoing safety assessments and in relation to WHO growth standards (21).

Infants were PCR-tested for HIV at birth, 10 weeks, and 6 months per the RSA standard of care (22). PCR tests were performed at well-baby clinics, with results provided to the clinical research site. The standard of care requires anti-HIV antibody testing at 18 months of age for all infants. To distinguish between vaccine-induced antibodies and HIV acquisition, infants had blood collected for testing via an HVTN-developed diagnostic algorithm at study month 17, with results provided to the local pediatrician. Infants were also tested via the HVTN diagnostic algorithm at the final visit at study month 24.5 to rule out HIV acquisition during the course of follow-up.

Sample collections from the infant participants were designed to maximize the scientific information obtained from small volumes, including exploratory systems vaccinology endpoints (e.g., transcriptomics, proteomics, and metabolomics), while ensuring the overall safety of the participants (16). This approach was complemented by routine monitoring of hemoglobin to monitor for anemia during the study.

### HVTN 135 trial: implementation

#### Site outreach to potential participants.

Potential participants were recruited by the Perinatal HIV Research Unit (PHRU) at Chris Hani Baragwanath Hospital in Johannesburg, South Africa. Two factors informed the recruitment strategy: first, the requirement for cesarean delivery (see *Study population* section above); and second, to identify birth parents who would potentially be able to follow the study schedule.

Since cesarean deliveries are not performed as standard of care for pregnant women living with HIV in RSA, in collaboration with the Chris Hani Baragwanath Academic Hospital (CHBAH) Obstetrics Department, individuals likely to undergo cesarean delivery (for example, with a previous cesarean delivery) were approached for participation in the study.

Recruitment was conducted by professional nurses who had the requisite knowledge and understanding to explain and answer questions about the study and any complex concepts. Possible barriers to retention were addressed by ascertainment of the pregnant woman’s current and future emotional and financial support network. With the mother’s consent, the nurses invited and engaged other individuals who formed part of the mother’s support system and were potentially involved with decisions regarding the future of the infant.

#### Maternal support.

The mothers had open access to the PHRU clinic during weekdays, with the ability to come to the clinic any time they needed. At enrollment they were provided with a 24-hour emergency number for one of the study doctors. During the hospital admission for delivery, the study team had daily contact with the mothers and the CHBAH Obstetrics team. Two on-call study team members at minimum were present at the delivery, often consisting of 2 nurses, or sometimes 1 nurse and 1 doctor. After the birth, the team had daily contact with the mother and the CHBAH staff caring for the infant.

A driver transported the mother and infant to and from the PHRU clinic for study visits during the first 2–4 weeks postpartum and whenever needed thereafter. On-site counseling services were made available for the mothers, which offered psychological support. Nutritional support and iron supplementation were provided as needed for the infants.

#### Navigating the COVID-19 pandemic.

The study enrolled participants during the COVID-19 pandemic between November 2020 and July 2022, representing an additional challenge to this complex study. The study team adhered to the PHRU and CHBAH COVID-19 policies designed to minimize risks to staff and participants. The protocol was amended to exclude birthing parents who tested positive for SARS-CoV-2 by PCR.

During periods of COVID-19 outbreaks in the country, changes in the CHBAH Obstetrics Department to mitigate risk and manage patient burden and staff illnesses included allocating dedicated COVID-19 wards and operating theaters and referring patients without COVID-19 to other hospitals for cesarean delivery. This meant that the study team on call had to be available 24 hours per day and had to follow up with participants in other hospitals, ensure equipment and laboratory kits were readily accessible, and organize 24-hour laboratory sample collection and processing.

### Statistics

Frequencies and proportions were calculated for categorical variables, whereas medians and interquartile ranges were determined for continuous measures. WHO growth standards (21) were used to interpret weight-for-age and weight-for-length *z* scores that were categorized into 3 groups: less than –2 (indicative of underweight for weight-for-age; wasting for weight-for-length), –2 to 2, and greater than 2. Participants were analyzed by randomization group, except that all placebo recipients and the 2 cohorts from parts B and C that received the same vaccine dose were combined for analysis. Statistical analysis was conducted using the open-source R program version 4.0.4 (https://www.R-project.org).

### Study approval

Conducting vaccine research in infants presents several challenging ethical issues related to parental permission, risks and benefits, and blood draw limits. The HVTN incorporated teams with expertise in neonatal immunity and systems vaccinology. The protocol included sample sparing assays, and the team conducted extensive stakeholder engagement with community representatives and with ethical and regulatory consultants during protocol development. The trial was overseen by the University of the Witwatersrand Human Research Ethics Committee, reference 190914B.

### Data availability

Data have been shared using the Atlas Science Portal (https://atlas.scharp.org/project/HVTN%20Public%20Data/begin.view).

## Author contributions

LP (DAIDS medical officer) assisted with the primary design of the study, was part of the oversight team, and contributed to writing and editing of the manuscript. VB contributed to the design and execution of the study. AV, OL, KKS, WH, GT, TMM, BH, NNM, JK, WBW, ZKS, MBI, KO, LC, HJ, and GG assisted with primary HVTN 135 study design. HJ and SC accessed and verified the underlying data. OL, AA, ASWD, KKS, TMM, HJ, and CY read the draft manuscript for detailed editorial feedback. LC approved the funding of the study, and assisted with study design, writing, review, and editing. AV, WH, HJ, KO, DJ, and SC drafted the manuscript.

## Supplementary Material

Supplemental data

ICMJE disclosure forms

Supporting data values

## Figures and Tables

**Table 2:**
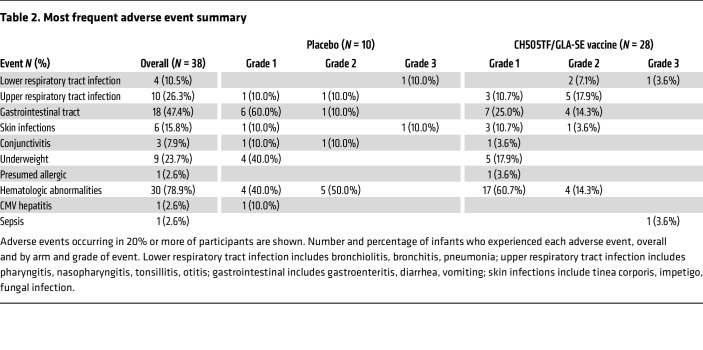
Most frequent adverse event summary

**Table 3:**
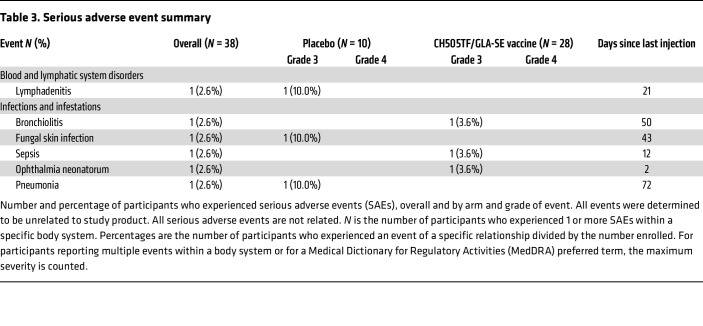
Serious adverse event summary

**Table 1:**
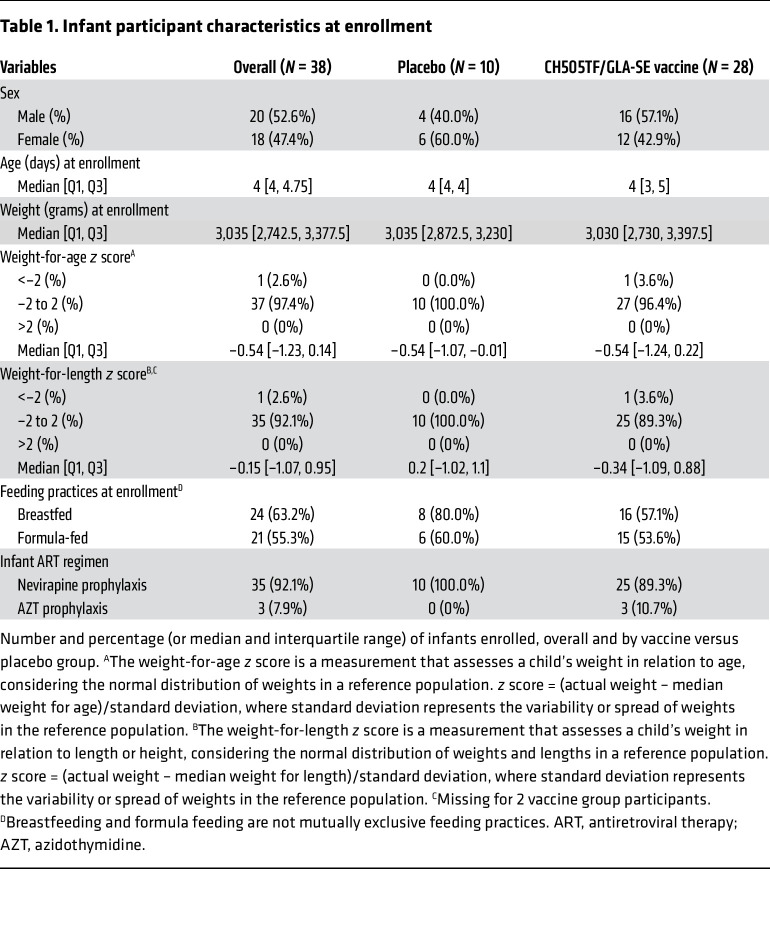
Infant participant characteristics at enrollment

## Version 1 04/03/2025

In-Press Preview

## Version 2 06/02/2025

Electronic publication
